# Malaria in Austria: A retrospective analysis of malaria cases diagnosed at a reference center in 2010–2020

**DOI:** 10.1007/s00508-023-02179-3

**Published:** 2023-04-17

**Authors:** Paul Horak, Herbert Auer, Ursula Wiedermann, Julia Walochnik

**Affiliations:** Institute of Specific Prophylaxis and Tropical Medicine, Medical University of Vienna, Kinderspitalgasse 15, 1090 Vienna, Austria

**Keywords:** Plasmodium, Imported, Travel medicine, Visiting friends and relatives (VFRs), Migration

## Abstract

**Background:**

Although malaria is not endemic to Austria, each year infections are imported by travellers, migrants and refugees. This study aims to provide an overview of malaria cases diagnosed at an Austrian institute for tropical medicine between 2010 and 2020.

**Methods:**

A retrospective, descriptive study was conducted based on the data of malaria cases confirmed at the Institute of Specific Prophylaxis and Tropical Medicine of the Medical University of Vienna. Laboratory diagnostics included microscopy, polymerase chain reaction (PCR) and real-time quantitative PCR.

**Results:**

Overall, 122 cases were identified. Annual case numbers were consistently higher from 2016 to 2020 than during the first half of the decade. Most malaria cases were diagnosed during summer and early autumn. This seasonal trend was not observed during the year 2020. With 55.1% (65/118) *Plasmodium falciparum* was the most common species, followed by *Plasmodium vivax* (19.5%, 23/118). The majority of patients were male (71.1%, 86/121) and the median age was 34.5 years (interquartile range, IQR 22.5–47.0 years). With a median age of 20.0 years (IQR 14.0–32.0 years), patients with *P. vivax* infections were younger than those infected with other *Plasmodium* species. Moreover, they were mostly male (82.6%, 19/23).

**Conclusion:**

From 2010 to 2020, the number of malaria cases diagnosed at the center increased. Growing international mobility and changing travel behavior could at least partly be responsible for this trend and there are indications that particularly* P. vivax* infections were imported by migrants and refugees.

## Introduction

Malaria is a parasitic disease caused by single-celled eukaryotic organisms of the genus *Plasmodium* and transmitted by the bite of female *Anopheles* mosquitos. The disease is prevalent in various subtropical and tropical regions around the world. Over the past two decades significant progress has been made in reducing the global malaria burden; however, malaria still ranks among the most important infectious diseases worldwide. In 2020, there were approximately 241 million malaria cases worldwide and about 627,000 deaths caused by the disease. Overall, 602,000 (96%) of all deaths occurred in Africa, and 77% of the total malaria fatalities were children under the age of 5 years [1].

The genus *Plasmodium *is a large taxonomic group with more than 250 known species that infect different vertebrate hosts [2]. Before the use of polymerase chain reaction (PCR) for malaria diagnosis, four species of *Plasmodium* were known to be pathogenic to humans: *Plasmodium falciparum*, *Plasmodium vivax*, *Plasmodium ovale* and *Plasmodium malariae*. Although primarily a parasite of long-tailed macaque monkeys in Southeast Asia, *Plasmodium knowlesi*, has been found to also infect humans [3]. Furthermore, *P. ovale* has been divided into two sympatric types, namely *P. ovale curtisi* and *P. ovale wallikeri*, only distinguishable by molecular methods [4, 5]. In terms of prevalence and severity of disease, *P. falciparum* and *P. vivax* are of highest importance, with the former being most likely to cause severe disease and death [6].

Europe has been widely free of autochthonous malaria transmission since 1975 [7]. The disease is reportable in most European countries, including Austria and today most registered cases are imported by international travellers, including particularly so-called visiting friends and relatives (VFRs), but also tourists and people traveling for work, and by migrants and refugees. According to data from the European Centre for Disease Prevention and Control (ECDC), from 2014 to 2018 there were between 7831 and 8427 annually reported malaria cases within the European Union [8].

A significant part of imported malaria cases in Europe occur in the group of VFR, who are often not aware that they lost their semi-immunity by living in Europe and thus have a lower risk perception [9]. In recent years, also migration and refugee movements have had an impact on the number of imported malaria cases in many European countries [10–12].

Austria received the status malaria-free by the World Health Organization (WHO) in 1963 [13]; however, just like in other European countries, malaria is regularly imported from endemic regions. Historically, the highest number of malaria cases were imported in the post-war period from 1946 to 1950. This was due to former soldiers returning from northern Africa, the Balkans, and the Mediterranean region. Between 1951 and 1975 much fewer cases were registered, usually less than 10 cases a year. Since then there has been a great increase in international tourism and case numbers have risen again [14]. A 2003 study on Austrian malaria cases found that from 1990 to 2000 there were on average 84 imported malaria cases per year, with *P. falciparum* responsible for 55.9% of all cases and no decreasing or increasing trends [15]. Since then, to the best of knowledge, there have been no further studies on this issue. The current study aimed to provide more up to date information on annual malaria cases and the involved *Plasmodium* species based on our diagnostic data.

## Patients, material and methods

A retrospective descriptive study was conducted based on the data of malaria cases confirmed at the Institute of Specific Prophylaxis and Tropical Medicine (ISPTM) of the Medical University of Vienna, the Austrian reference center for parasitic infections. While the Institute receives samples from healthcare centers from all federal states of the country, the majority come from eastern Austria with approximately 60.0% of the study samples coming from healthcare centers in Vienna. A laboratory quality assurance program with regular international interlaboratory tests ensures high levels of accuracy and proficiency. A case was defined as any individual with direct proof of an infection with one or more species of *Plasmodium* between 1 January 2010 and 31 December 2020.

Laboratory diagnostics included light microscopy (Giemsa stained thin and thick blood smears), polymerase chain reaction (PCR), real-time quantitative PCR (qPCR), rapid diagnostic tests (RDT) (BinaxNOW® Malaria, Abbott Rapid Diagnostics Austria GmbH, Linz, Austria) or a combination of these. A standard nested PCR protocol [16] has been in routine clinical use at the ISPTM since 2014: blood samples with a positive *Plasmodium* genus-specific reaction were tested with species-specific nested PCRs, differentiating between *P. falciparum, P. vivax, P. ovale curtisi, P. ovale wallikeri* and *P. malariae*. Samples that were positive in the genus-specific reaction but had negative species-specific results were additionally tested for *P. knowlesi*, following published protocols [17, 18]. A novel fluorescence resonance energy transfer-based real-time PCR (FRET-qPCR) was introduced in the year 2020 and was used in addition to the nested PCR. This novel assay reportedly has a higher sensitivity than the standard nested PCR protocol, combined with faster turnaround times [19].

During the observed time period, blood samples of more than 600 patients underwent direct pathogen detection for *Plasmodium* spp. During the course of the study period there was a marked increase in patient samples that the institution received for malaria diagnostics, with almost two thirds of all patients tested between 2016 and 2020. All patients were anonymized and results from each type of diagnostic test (microscopy, genus-specific PCR, species-specific PCR, FRET-qPCR, RDT) were downloaded into Microsoft Excel® (Microsoft Corporation, Redmond, WA, USA) files. Data were filtered for positive results and all double entries were removed. Altogether, 122 cases were identified and included. Many patients were diagnosed using various test methods, and in a few cases, the results concerning the species of *Plasmodium *(usually *P. vivax *and *P. ovale*) did not match between microscopy and PCR. With respect to the higher sensitivity and specificity of molecular diagnostic methods, PCR-based results were used in such cases.

Parameters evaluated were time of diagnosis, gender, age and *Plasmodium* species identified. In order to evaluate seasonality of malaria in Austria, cumulative monthly case numbers of the entire time period were summarized. To evaluate impacts of the coronavirus disease 2019 (COVID-19) pandemic, monthly case numbers were also analyzed separately for the year 2020. Additionally, demographic characteristics (gender, age) of patients were analyzed for each *Plasmodium* spp.

All parameters were described using descriptive statistics, without further statistical tests. To perform statistical analyses and create graphs, the programs Microsoft Excel® and IBM SPSS Statistics 27® (IBM Corporation, Armonk, NY, USA) were used.

## Results

**Fig. 1:**
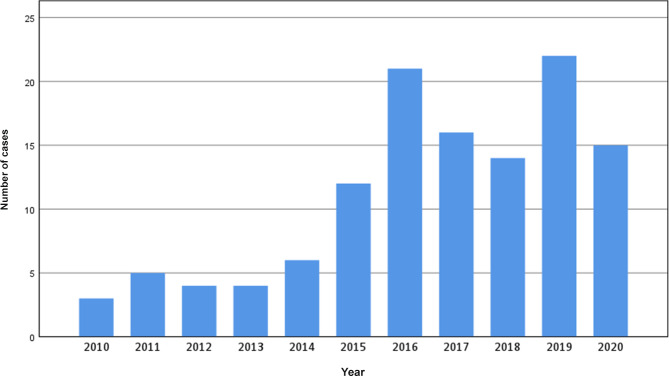
Number of annual malaria cases diagnosed at the Institute of Specific Prophylaxis and Tropical Medicine (ISPTM) of the Medical University of Vienna (2010–2020)

Within the observed time period 122 malaria cases were diagnosed at our institution, with an annual mean of 11.1 cases. Absolute frequencies of annual malaria cases are given in Fig. 1.

The lowest number of cases (2.5%, 3/122) was observed in 2010. From 2011 to 2014 case numbers were relatively low, with 4 (3.3%, 4/122) to 6 (4.9%, 6/122) cases per year, subsequently rising to 12 (9.8%, 12/122) cases in 2015 and 21 (17.2%, 21/122) cases in 2016. With 16 (13.1%, 16/122) and 14 (11.5%, 14/122) cases a slight decrease in numbers could be observed in the years 2017 and 2018, before a maximum of 22 (18.0%, 22/122) cases was reached in 2019, followed by 15 (12.3%, 15/122) cases in 2020.

Most cases were reported from June to September, with 19 (15.6%, 19/122) cases in August, 16 (13.1%, 16/122) in September, 13 (10.7%, 13/122) in June and 12 (9.8%, 12/122) in July. January and March each had 10 (8.2%, 10/122) cases, and there were 9 (7.4%, 9/122) cases in October, 8 (6.6%, 8/122) in May, 7 (5.7%, 7/122) in both April and December and 6 (4.9%, 6/122) in November. With 5 cases (4.1%, 5/122) the lowest number was registered in February.

In 2020, the majority of cases were reported from January to March. There were 3 (20.0%, 3/15) cases in both January and February, followed by 5 (33.3%, 5/15) cases in March. During the rest of the year, cases were only reported sporadically: one (6.7%, 1/15) individual each, was diagnosed in the months July, September, October and December.

Of the 122 positive cases, the gender and age of one patient could not be determined as these data were missing from the dataset. With 71.1% (86/121) the majority of cases were male, while 28.9% (35/121) were female. The median age was 34.5 years with an interquartile range (IQR) of 22.5–47.0 years. The youngest person was 1 year of age, while the oldest was 84 years old.

**Fig. 2:**
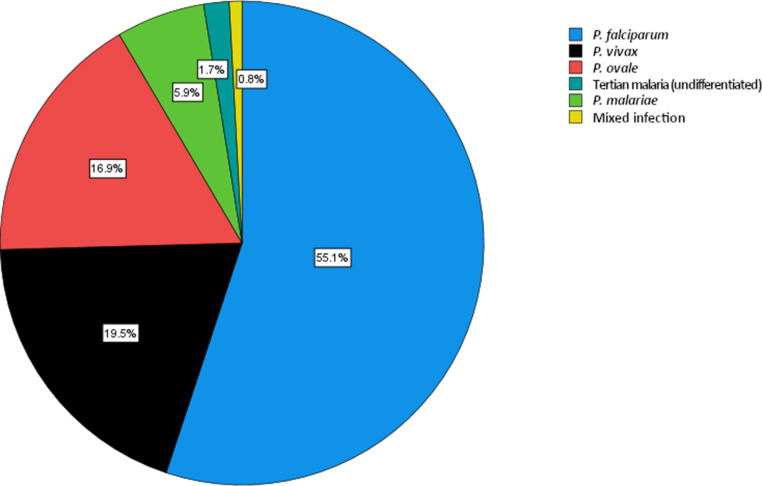
Relative frequency of malaria cases sorted by species from patients diagnosed at the ISPTM of the Medical University of Vienna (2010–2020)

Relative frequencies of the involved *Plasmodium *spp. are shown in Fig. 2. In four cases, the species could not be determined. The majority of individuals were diagnosed with *P. falciparum *(55.1%, 65/118), followed by *P. vivax* (19.5%, 23/118) and *P. ovale* (16.9%, 20/118). There were two (1.7%, 2/118) cases of undifferentiated tertian malaria. The overall share of patients with tertian malaria was therefore 38.1% (45/118). Infections with *P. malariae* were much less common (5.9%, 7/118) and one (0.8%, 1/118) patient had a mixed infection with both *P. falciparum* and *P. ovale*.

*P. ovale* was detected in 21 individuals, including 1 mixed infection. A differentiation between *P. ovale curtisi* and *P. ovale wallilkeri* by PCR was made in 15 of these cases. *P. ovale curtisi* was more common and could be detected in 60.0% (9/15) of cases. *P. ovale wallikeri* was responsible for the remaining 40.0% (6/15) of cases.

**Fig. 3:**
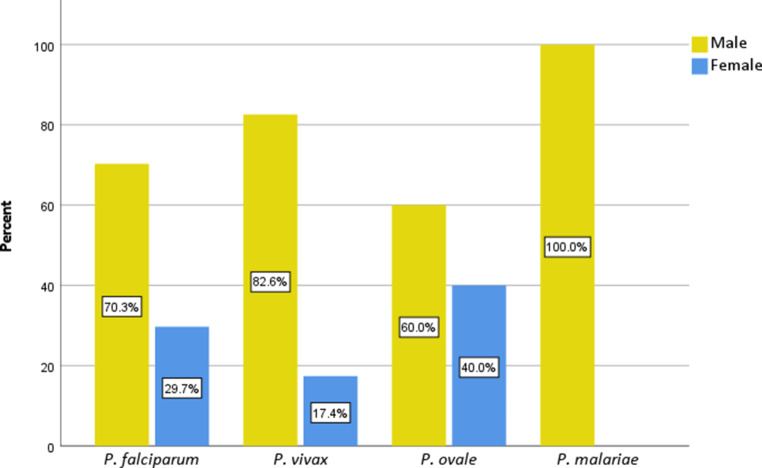
Patient sex in relation to *Plasmodium* species (spp.) of patients diagnosed at the ISPTM of the Medical University of Vienna (2010–2020)

**Fig. 4:**
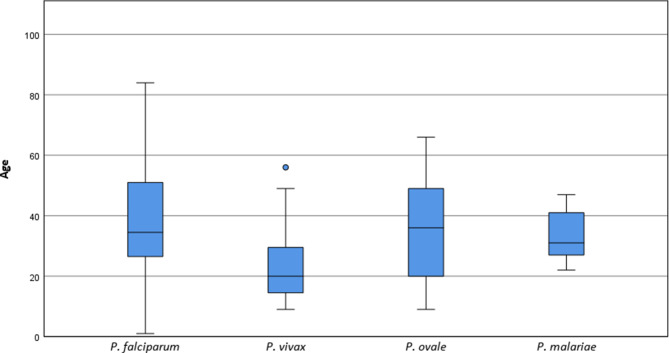
Box and whisker plots of patient age (in years) in relation to *Plasmodium *spp. of patients diagnosed at the ISPTM of the Medical University of Vienna (2010–2020)

Sex ratios were established in relation to the involved *Plasmodium* spp. (Figure 3) and the median age of patients was calculated for each species (Fig. 4). For the 114 included cases, sufficient data on *Plasmodium *spp. and gender and age of the respective patient were available. Undifferentiated tertian malaria cases and mixed infections were not included. For *P. falciparum*, 70.3% (45/64) of cases were male, while 29.7% (19/64) were female. Of the patients infected with *P. vivax*, 82.6% (19/23) were male and 17.4% (4/23) were female. Sex ratio was more balanced in *P. ovale* cases, with 60.0% (12/20) being male and 40.0% (8/20) female. All seven patients with *P. malariae* infections were male (100.0%, 7/7).

The median age for *P. falciparum* cases (*n* = 64) was 34.5 years with an IQR of 26.3–52.0 years. *P. vivax* cases (*n* = 23) had a median age of 20.0 years (IQR 14.0–32.0 years), while the median age for individuals infected with *P. ovale *(*n* = 20) was 36.0 years (IQR 19.5–51.0 years). Patients who had an infection with *P. malariae *(*n* = 7) had a median age of 31.0 years and an IQR of 26.0–42.0 years.

## Discussion

This study evaluated and described malaria cases diagnosed at the ISPTM of the Medical University of Vienna between 1 January 2010 and 31 December 2020. In total, 122 cases were recorded at our institution, which relates to about one fifth of all malaria cases reported in Austria during this time period (595 cases) [21, 22]. Results suggest an increasing trend in case numbers. The majority of patients were male (71.1%) and the median age was 34.5 years. With 55.1% *P. falciparum* was the most common species, followed by *P. vivax* (19.5%).

Although malaria case numbers worldwide have, for the most part, been declining since the turn of the millennium [20], this is not the case in non-endemic regions. At our institution, annual case numbers were consistently higher in the years 2016–2020 than from 2010–2015, with a peak in case numbers in the year 2019; however, as there was an increase in patient samples that our institution received over time, these results could rather reflect an increase in conducted tests than an actual increase in cases. In most European countries, including Austria, malaria is a notifiable disease, thus official national data are available. To better assess the overall development of case numbers within the entire country, in the following we provide absolute and relative frequencies of malaria cases using data from Austria’s national reporting system [21].

In 2010, there were 48 (8.0%, 48/595) cases, followed by 7 (1.2%, 7/595) cases in 2011, 28 (4.7%, 28/595) cases in 2012 and 42 (7.1%, 42/595) cases in 2013. During the next years case numbers increased, from 68 (11.4%, 68/595) cases in 2014 to 81 (13.6%, 81/595) cases in 2015 and a maximum of 82 (13.8, 82/595) cases in 2016. Numbers remained relatively high with 78 (13.1%, 78/595), 62 (10.4%, 62/595) and 72 (12.1%, 72/595) cases in the years 2017, 2018 and 2019, respectively, before finally dropping to 27 (4.5%, 27/595) cases in 2020. Official data therefore showed a similar pattern on case numbers as data from our institution, with the number of cases increasing in the second part of the decade, reaching a peak in 2015 and 2016 [21]. This trend was also seen in the entire European Union, where the peak was reached in 2019 with 8638 total cases (in 2020: 2321 cases) [22].

Apart from confounding factors, such as increased testing, also growing international mobility, altered travel behavior and migration movements might be responsible for the overall increase in case numbers. All of these developments were also taking place in Austria, with the past decade holding a significant influx of migrants and refugees, reaching a maximum of 88,340 asylum applications in the year 2015 [23]. Moreover, both outbound and inbound tourism increased from 2010 to 2019, as data from the World Tourism Organization (UNWTO) suggests [24]. A factor contributing to a rise in malaria infections among tourists could be an increased incidence of more “careless travelling”, as malaria often occurs in travellers who do not take chemoprophylaxis or use it inadequately [25, 26]. In contrast to the early years of tropical tourism, today, many travellers do not seek pretravel consultation at all, most often because of low risk perception [27], which is already known from the VFR group, as described above. Vygen-Bonnet and Stark reported a decline of chemoprophylaxis usage among imported malaria cases in Germany between 2001 and 2016 [10]. The majority of travellers make their bookings online [28], which, while being convenient and quick, might be counterproductive when it comes to encouraging individuals to inform themselves about health concerns prior to travelling. Even if chemoprophylaxis is prescribed, rates of non-compliance between 11% and 38% have been reported [29], with common reasons for non-adherence being “not seeing mosquitoes”, forgetfulness and medication side effects [30].

When analyzing seasonality, it was shown that most cases were diagnosed during summer and early autumn, especially in the months of August (15.6%, 19/122) and September (13.1%, 16/122). These findings are in accordance with data from the rest of Europe [22]. Seasonality is most likely related to the summer holiday season, which comes hand in hand with more people travelling abroad; however, due to the COVID-19 pandemic and its accompanying travel restrictions, international tourism came to a rather abrupt and involuntary halt in the year 2020. International arrivals dropped by 74%, with one billion fewer arrivals than in the previous year [31]. When taking a closer look at the year 2020, it becomes apparent that the majority of cases (73.3%, 11/15) were observed between January and March. With Austria’s first lockdown starting on 16 March 2020, most patients were therefore diagnosed before and at the beginning of the first coronavirus wave. During the rest of the year, with travel restrictions now largely implemented, only four more cases were diagnosed, one each in July, September, October and December. The aforementioned seasonal patterns could therefore not be observed in the year 2020.

Interestingly, the majority of cases were male (71.1%, 86/121). This finding is also in accordance with data from other European countries and European surveillance [10, 30, 32]. The male predominance might be explained by Austrian travel patterns, with potentially more men travelling to the tropics. Moreover, the majority of migrants and refugees were male [23]. Some studies have also suggested gender-specific differences in seeking pretravel consultation and adhering to chemoprophylaxis [33, 34]. Men are likely to take more risks while travelling (e.g. engage in outdoor activities, sleep in tents, etc.) and to travel to more remote, rural areas with a higher risk of acquiring malaria [35]; however, malaria was not restricted to a certain age class. Patients were aged between 1 and 84 years, with a median age of 35 years. A 2015 analysis of European surveillance data reported the same median age for patients presenting with any travel-associated illnesses in general [36]. Therefore, this result is probably a reflection of the age group in the Austrian population that is most likely to travel to tropical regions, particularly with a travel behavior that implies a higher risk to acquire vector-borne infections. It is also in accordance with the young age of many migrants and refugees [37]. Of note, most cases were adults, which is in stark contrast to endemic countries, where the main burden of malaria morbidity and mortality is carried by children under the age of 5 years [38].

With 55.1% (65/118), *P. falciparum* was by far the most common species. This was of little surprise, as* P. falciparum* has the highest prevalence of all malaria parasites worldwide [38] and is also the most likely species to cause severe disease [39], thus rarely remaining undiagnosed; however, *P. falciparum* was less common than one would have assumed when looking at European surveillance data, for example the ECDC reported a frequency of 88.2% for this species in the year 2019 [22]. In the current study 38.1% (45/118) of patients had tertian malaria, with *P. vivax* being a little more common than *P. ovale*. PCR was used to discriminate between *P. ovale wallikeri* and *P. ovale curtisi *infections and it was shown that *P. ovale curtisi* was more common (60.0%, 9/15) than *P. ovale wallikeri*; however, conclusions are limited by the very small case number (*n* = 15). Nevertheless, they are in accordance with data from returning travellers in Italy [40] and China [41], which also showed higher numbers of *P. ovale curtisi *cases. Additionally, a 2021 meta-analysis comparing the two types, found a significantly higher proportion of *P. ovale curtisi* cases [42]. Other forms of malaria were rare: only seven (5.9%, 7/118) individuals were diagnosed with *P. malariae*. The species is believed to have a somewhat patchy geographic distribution and to only occur infrequently in areas of endemicity [43]. It seems likely that tourists simply are only very rarely infected with this parasite. Of note, all cases infected with *P. malariae* were male. The cause for this remains unclear, but it might just be coincidence and attributed to the small case number (*n* = 7). The rarity of *P. malariae* cases was exceeded only by *P. knowlesi* infections of which there were none. This can probably be attributed to the infrequency of *P. knowlesi* infections in humans and a very limited geographic distribution of the species [44]. Apart from that, *P. knowlesi* is also difficult to diagnose by light microscopy and probably suffers from underreporting [45]; however, since the introduction of PCR into standard routine malaria diagnostics at the ISPTM in 2014, *P. knowlesi* cases would likely have been identified, if there had been any [19]. It was revealed that 82.6% (19/23) of individuals with a *P. vivax* infection were male, a higher percentage than for *P. falciparum* (70.3%, 46/64) and *P. ovale* (60.0%, 12/20). Furthermore, those infected with *P. vivax* were on average younger than those infected with other *Plasmodium* spp.: the median age was just 20.0 years, while the median age for patients infected with other species ranged from 31.0 to 36.0 years. The demographic data of *P. vivax* cases thus had apparent similarities to those of migrants and refugees, of which the majority were also young and male [23]. Therefore, it seems plausible that a significant proportion of these infections were imported by this population group. This hypothesis is also supported by the origin of migrants and refugees arriving in Austria: from 2014 to 2017, approximately half originated either from Syria or Afghanistan [23]. According to the WHO, Syria has not reported any indigenous malaria cases since 2004 [38], but due to the ongoing civil war, data available on malaria transmission in the country are limited; however, malaria is endemic to Afghanistan, with the predominant species being *P. vivax *[38, 46]. While in the current study, geographic data was only available for less than a third of all cases, Afghanistan was the most frequently named non-African country.

## Conclusion

From 2010 to 2020, the number of malaria cases diagnosed at the Institute of Specific Prophylaxis and Tropical Medicine (ISPTM) of the Medical University of Vienna increased, probably at least partly due to international travel, migration and refugee movements. A seasonal pattern with more cases during the summer months was observed, with the exception of the year 2020. It can be assumed that this was due to a decrease in international mobility caused by the coronavirus disease 2019 (COVID-19) pandemic and its accompanying travel restrictions. Although the origin of cases was not evaluated in this study, demographic data of patients infected with *Plasmodium vivax* suggest that *P. vivax* cases were closely linked to migrants and refugees arriving in Europe in 2015 and subsequent years. Therefore, clinicians have to be aware of the fact that individuals with respective backgrounds presenting with febrile illness could potentially have malaria; however, even in the absence of migration and refugee movements, cases of imported malaria will likely continue to increase as it becomes simpler and easier for travellers to reach far-off destinations without taking the essential travel preparations. This study provides valuable epidemiological data for future research and surveillance.

## References

[1] World Health Organization. World malaria report. 2021. https://www.who.int/teams/global-malaria-programme/reports/world-malaria-report-2021. Accessed 15 July 2022.

[2] Ramasamy R (2014). Zoonotic malaria—global overview and research and policy needs. Front Public Health.

[3] Singh B, Kim Sung L, Matusop A (2004). A large focus of naturally acquired plasmodium knowlesi infections in human beings. Lancet.

[4] Zaw MT, Lin Z (2017). Two sympatric types of plasmodium ovale and discrimination by molecular methods. J Microbiol Immunol Infect.

[5] Sutherland CJ, Tanomsing N, Nolder D (2010). Two nonrecombining sympatric forms of the human malaria parasite plasmodium ovale occur globally. J Infect Dis.

[6] Phillips MA, Burrows JN, Manyando C, van Huijsduijnen RH, Van Voorhis WC, Wells TNC (2017). Malaria. Nat Rev Dis Primers.

[7] World Health Organization. History of malaria elimination in the European Region. https://www.euro.who.int/__data/assets/pdf_file/0003/307272/Facsheet-malaria-elimination.pdf. Accessed 9 Oct 2020.

[8] European Centre for Disease Prevention and Control. Malaria—annual epidemiological report for 2018. https://www.ecdc.europa.eu/en/publications-data/malaria-annual-epidemiological-report-2018. Accessed 29 June 2021.

[9] Tatem AJ, Jia P, Ordanovich D (2017). The geography of imported malaria to non-endemic countries: a meta-analysis of nationally reported statistics. Lancet Infect Dis.

[10] Vygen-Bonnet S, Stark K (2018). Changes in malaria epidemiology in Germany, 2001–2016: a time series analysis. Malar J.

[11] Sondén K, Castro E, Trönnberg L, Stenström C, Tegnell A, Färnert A (2014). High incidence of plasmodium vivax malaria in newly arrived Eritrean refugees in Sweden since May 2014. Euro Surveill.

[12] Eperon G, Durieux-Paillard S, Mauris A, Chappuis F, Gysin N (2017). Malaria cases in Switzerland from 2005 to 2015 and recent rise of imported plasmodium vivax malaria. Swiss Med Wkly.

[13] World Health Organization. Countries and territories certified malaria-free by WHO. http://www.who.int/malaria/areas/elimination/malaria-free-countries/en/. Accessed 12 Oct 2020.

[14] Ambrosch F, Halbich H (1985). Malaria-Einschleppungen in Österreich. Mitt Osterr Ges Tropmed Parasitol.

[15] Strauss R, Pfeifer C (2003). Malaria in Austria 1990–2000. Euro Surveill.

[16] Snounou G, Clapp JP (1996). Detection and identification of the four malaria parasite species infecting humans by PCR amplification. Species diagnostics protocols: PCR and other nucleic acid methods.

[17] Singh B, Bobogare A, Cox-Singh J, Snounou G, Abdullah MS, Rahman HA (1999). A genus- and species-specific nested polymerase chain reaction malaria detection assay for epidemiologic studies. Am J Trop Med Hyg.

[18] Imwong M, Tanomsing N, Pukrittayakamee S, Day NPJ, White NJ, Snounou G (2009). Spurious amplification of a plasmodium vivax small-subunit RNA gene by use of primers currently used to detect P. knowlesi. J Clin Microbiol.

[19] Schneider R, Lamien-Meda A, Auer H, Wiedermann-Schmidt U, Chiodini PL, Walochnik J (2021). Validation of a novel FRET real-time PCR assay for simultaneous quantitative detection and discrimination of human plasmodium parasites. PLoS ONE.

[20] World Health Organization. World malaria report. 2019. https://www.who.int/publications-detail-redirect/9789241565721. Accessed 24 Oct 2020.

[21] Österreichisches Sozialministerium. Übertragbare Krankheiten. https://www.sozialministerium.at/Themen/Gesundheit/Uebertragbare-Krankheiten/Statistiken-und-Fallzahlen.html. Accessed 14 Oct 2020.

[22] European Centre for Disease Prevention and Control. Malaria—annual epidemiological report for 2019. https://www.ecdc.europa.eu/en/publications-data/malaria-annual-epidemiological-report-2019. Accessed 22 Apr 2021.

[23] Österreichisches Bundesministerium für Inneres. Statistiken Asyl. https://www.bmi.gv.at/301/Statistiken/. Accessed 14 Sept 2021.

[24] World Tourism Organization. Tourism statistics—country fact sheets. https://www.unwto.org/statistics/country-fact-sheets. Accessed 30 Aug 2021.

[25] Vliegenthart-Jongbloed K, de Mendonça Melo M, van Wolfswinkel ME, Koelewijn R, van Hellemond JJ, van Genderen PJJ (2013). Severity of imported malaria: protective effect of taking malaria chemoprophylaxis. Malar J.

[26] Jelinek T, Schade Larsen C, Siikamäki H (2008). European cluster of imported falciparum malaria from Gambia. Euro Surveill.

[27] Kain D, Findlater A, Lightfoot D (2019). Factors affecting pre-travel health seeking behaviour and adherence to pre-travel health advice: a systematic review. J Travel Med.

[28] Travel Weekly. Consumer trends 2014: the growing influence of tripadvisor. https://www.travelweekly.com/Travel-News/Online-Travel/The-growing-influence-of-TripAdvisor. Accessed 15 Aug 2022.

[29] Hoefnagel JGM, Massar K, Hautvast JLA (2020). Non-adherence to malaria prophylaxis: the influence of travel-related and psychosocial factors. J Infect Public Health.

[30] Stoney RJ, Chen LH, Jentes ES (2016). Malaria prevention strategies: adherence among boston area travelers visiting malaria-endemic countries. Am J Trop Med Hyg.

[31] World Tourism Organization. 2020: worst year in tourism history with 1 billion fewer international arrivals. https://www.unwto.org/news/2020-worst-year-in-tourism-history-with-1-billion-fewer-international-arrivals. Accessed 26 Aug 2021.

[32] Zanotti P, Odolini S, Tomasoni LR (2018). Imported malaria in northern Italy: epidemiology and clinical features observed over 18 years in the teaching hospital of Brescia. J Travel Med.

[33] Pavli A, Silvestros C, Patrinos S, Maltezou HC (2015). Vaccination and malaria prophylaxis among Greek international travelers to Asian destinations. J Infect Public Health.

[34] Schlagenhauf P, Chen LH, Wilson ME (2010). Sex and gender differences in travel-associated disease. Clin Infect Dis.

[35] Stienlauf S, Segal G, Sidi Y, Schwartz E (2005). Epidemiology of travel-related hospitalization. J Travel Med.

[36] Schlagenhauf P, Weld L, Goorhuis A (2015). Travel-associated infection presenting in Europe (2008–12): an analysis of EuroTravNet longitudinal, surveillance data, and evaluation of the effect of the pre-travel consultation. Lancet Infect Dis.

[37] Eurostat. Data explorer. https://appsso.eurostat.ec.europa.eu/nui/submitViewTableAction.do. Accessed 11 Sept 2021.

[38] World Health Organization. World malaria report. 2020. https://www.who.int/teams/global-malaria-programme/reports/world-malaria-report-2020. Accessed 28 June 2021.

[39] No authors listed (2014). Severe malaria. Trop Med Int Health.

[40] Calderaro A, Piccolo G, Gorrini C (2012). A new real-time PCR for the detection of plasmodium ovale wallikeri. PLoS One.

[41] Chen M, Dong Y, Deng Y (2020). Polymorphism analysis of propeller domain of k13 gene in plasmodium ovale curtisi and plasmodium ovale wallikeri isolates original infection from Myanmar and africa in Yunnan province, China. Malar J.

[42] Mahittikorn A, Masangkay FR, Kotepui KU, Milanez GDJ, Kotepui M (2021). Comparison of plasmodium ovale curtisi and plasmodium ovale wallikeri infections by a meta-analysis approach. Sci Rep.

[43] Mueller I, Zimmerman PA, Reeder JC (2007). Plasmodium malariae and plasmodium ovale—the ‘bashful’ malaria parasites. Trends Parasitol.

[44] Müller M, Schlagenhauf P (2014). Plasmodium knowlesi in travellers, update 2014. Int J Infect Dis.

[45] Lee KS, Cox-Singh J, Singh B (2009). Morphological features and differential counts of plasmodium knowlesi parasites in naturally acquired human infections. Malar J.

[46] Leslie T, Nahzat S, Sediqi W (2016). Epidemiology and control of plasmodium vivax in Afghanistan. Am J Trop Med Hyg.

